# Comparative analysis of external locking plate and combined frame external fixator for open distal tibial fractures: a comprehensive assessment of clinical outcomes and financial implications

**DOI:** 10.1186/s12891-023-07097-z

**Published:** 2023-12-11

**Authors:** Mohamed Lamin Bangura, Huasong Luo, Teng Zeng, Minglu Wang, Shangce Lin, Liang Chunli

**Affiliations:** 1 Department of Orthopedics, The First People’s Hospital of Jingzhou, The First Affiliated Hospital of Yangtze University, Jingzhou City, Hubei Province 434023 People’s Republic of China; 2 School of Medicine, Yangtze University, Jingzhou, China

**Keywords:** Locking compression plate, Unilateral External fixator, Combined frame external fixator

## Abstract

**Background:**

Open distal tibial fractures pose significant challenges regarding treatment options and patient outcomes. This retrospective single-centre study aimed to compare the stability, clinical outcomes, complications, and financial implications of two surgical interventions, namely the external locking plate and the combined frame external fixator, to manage open distal tibial fractures.

**Methods:**

Forty-four patients with distal open tibial (metaphyseal extraarticular) fractures treated between 2020 and 2022 were selected and formed into two main groups, Group A and Group B. Group A (19 patients) are patients that underwent treatment using the external locking plate technique, while Group B (25 patients) received the combined frame external fixator approach. Age, gender, inpatient stay, re-operation rates, complications, functional recovery (measured by the Johner-Wrush score), pain ratings (measured by the Visual Analogue Scale [VAS]), and cost analyses were evaluated for each group. Statistical analyses using SPSS were conducted to compare the outcomes between the two groups.

**Results:**

The research found significant variations in clinical outcomes, complications, and cost consequences between Group A and Group B. Group A had fewer hospitalisation periods (23.687.74) than Group B (33.5619.47). Re-operation rates were also considerably lower in Group A (26.3%) than in Group B (48%), owing to a greater prevalence of pin-tract infections and subsequent pin loosening in the combination frame external fixator group. The estimated cost of both techniques was recorded and analysed with the locking average of 26,619.69 ± 9,602.352 and the combined frame average of 39,095.64 ± 20,070.077.

**Conclusion:**

This study suggests that although the two approaches effectively manage open distal tibia fractures, the locking compression plate approach (Group A) has an advantage in treating open distal tibia fractures. Shorter hospitalisation times, reduced re-operation rates, and fewer complications will benefit patients, healthcare systems, and budget allocation. Group A's functional recovery results demonstrate the locking plate technique's ability to improve recovery and patient quality of life. According to the cost analysis, the locking plate technique's economic viability and cost-effectiveness may optimise healthcare resources for open distal tibia fractures. These findings might improve patient outcomes and inform evidence-based orthopaedic surgery.

**Supplementary Information:**

The online version contains supplementary material available at 10.1186/s12891-023-07097-z.

## Introduction

Open distal tibial fractures are challenging injuries with potential long-term complications. Although intramedullary (IM) nailing is widely used to treat closed tibial fractures, managing open distal type III tibial fractures with severe soft tissue damage or compartment syndrome can be challenging [1, 2]. The IM was initially crafted for the temporary management of open or closed fractures accompanied by significant soft tissue injuries, which heightened the likelihood of postoperative infections. Their usage has progressively expanded to encompass the less invasive treatment of closed fractures without soft tissue damage, given that reaming was unnecessary and the supply of blood to the inner bone region was less disrupted. Nevertheless, this approach has been associated with an elevated incidence of implant failure and subsequent revision surgeries [3]. A study conducted by Bakshi et al. found that the antibiotic-coated intramedullary interlocking nail (ACIIN) does not establish stability for bone defects over 4 cm. Instead, external fixators like Ilizarov and LRS should be employed. Antibiotic-coated nails, even though they were easier to use and prevented pin loosening and infections, showed that external fixators (Limb Reconstruction System/Ilizarov) stiffened neighbouring joints more than ACIIN [4]. External fixation can provide initial stability and allow for soft tissue damage monitoring before transitioning to definitive surgical reconstruction in such cases.

To achieve the best treatment outcomes in complex cases involving severe polytrauma, a staged approach involving external fixation for initial stabilisation followed by definitive reconstruction with IM may be required [5]. Recently, external fixators have been used as the definitive treatment for open distal tibial fractures. However, selecting the proper external fixation system is critical because it can significantly impact clinical outcomes. The combined frame external fixation system (external fixation bracket) and locking plates are two commonly used external fixation systems for open distal tibial fractures. Although the combined frame external fixation system has been used to treat these fractures with compromised soft tissue damage, its configuration is usually bulky and burdensome for patients [6, 7]. On the other hand, because of their low profile and angular stability, locking plates are preferred by many surgeons as an alternative external fixator for treating open tibial fractures [8, 9].

Can the locking plate better replace the combined frame's external fixation? Should the financial implications be a determinant in selecting the type of fixation to be used? Given this dilemma and distinctions, this study aimed to compare the stability, clinical outcomes, complications, and possible financial implications of using an external locking plate versus a combined frame external fixator in treating open distal tibial fractures. By examining the benefits and disadvantages of each system, clinicians can make more informed decisions when choosing an appropriate external fixation system for distal tibial fractures, improving patient outcomes.

## Materials and methods

Our hospital's ethics committee approved this retrospective study for medical research, and informed consent was obtained from all patients included in the study. We selected forty-four patients treated with distal open tibial (metaphyseal extraarticular) fractures between January 2019 and March 2022. Based on their treatment, the patients were divided into two main groups: Group A and Group B. Group A consisted of 19 patients who underwent treatment using the external locking plate technique. Group B included 25 patients who received the combined frame external fixator approach.

Several variables were evaluated for each group, including age, gender, inpatient stay, re-operation rates, complications, functional recovery (measured by the Johner-Wrush score), pain ratings (measured by the Visual Analogue Scale [VAS]), and cost analyses. Statistical analyses using SPSS were conducted to compare the outcomes between the two groups.

The inclusion criteria for this study were as follows: (1) patients who were 18 years or older; (2) patients who provided consent for participation; (3) patients admitted to Jingzhou People's First Hospital in Jingzhou, Hubei, China; (4) patients who underwent surgery for open distal tibial fractures; (5) patients with poor soft tissue conditions, including fractures with extensive skin abrasions, necrosis, localised multiple blisters, or Gustilo type I open fractures; (6) patients with Gustilo Type II/III open fractures with soft tissue defects; and (7) patients who accepted external fixation or cooperated with follow-up. The exclusion criteria were as follows: (1) patients under the age of 18, (2) patients with closed fractures, (3) patients with other medical conditions such as diabetes and osteoporosis, and (4) patients with insufficient follow-up. All surgeries were performed by the same surgeon, trained in both techniques.

### Surgery procedure

#### A. The locking steel plate external group

**Fig. 1:**
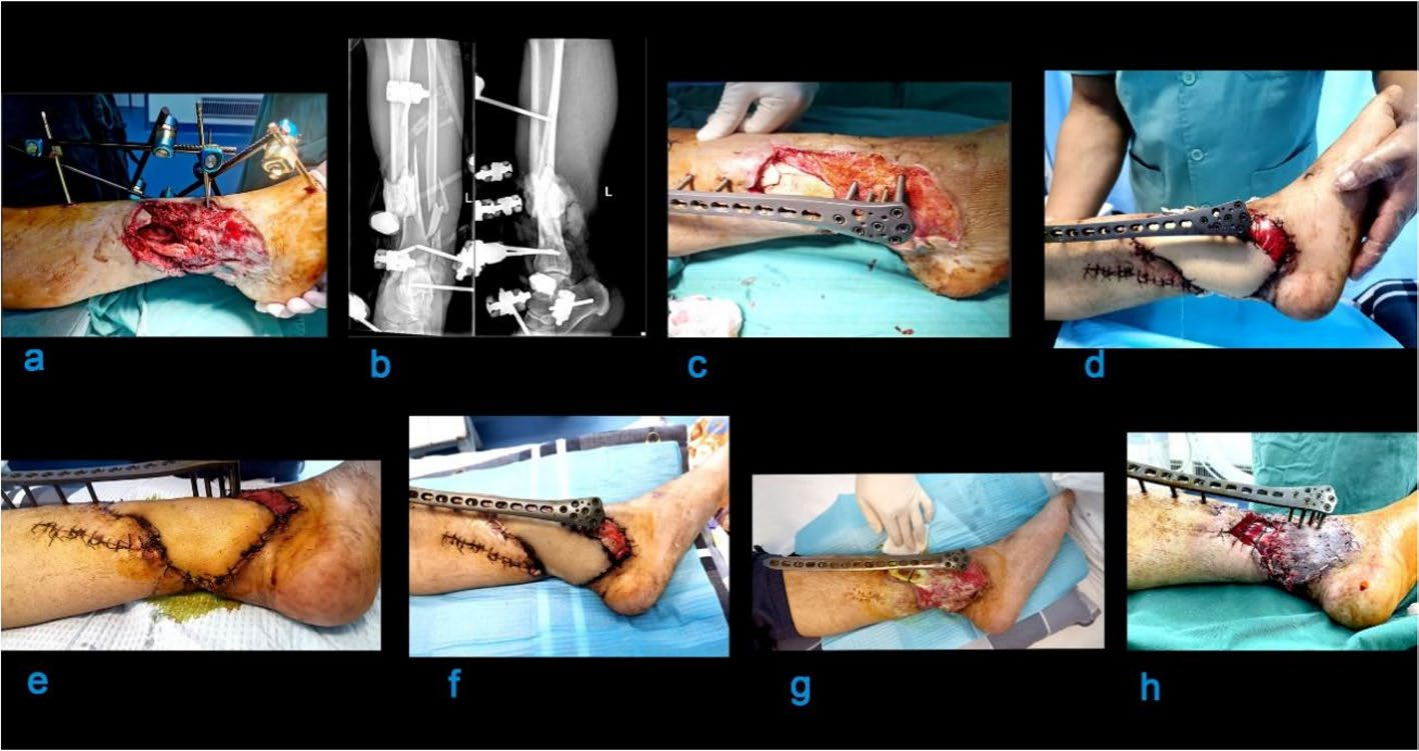
A 44 years old male patient with comminuted fracture (**a**) preoperative, (**b**) a computed tomography before the external fixator was replaced with the locking plate external fixator, (**c**) after repeated debridement, the external fixator was replaced with a locking plate external fixator, and the infection was controlled after VSD continues negative pressure drainage, (**d**) day one post-operation, (**e**) the 3rd-day post-operation, (**f**) sixth-day post-operation, (**g**) after re-vascular exploration, the skin flap gradually become necrotic, skin grafting was again performed, and repaired the local adjacent skin flap, (**h**) two weeks after the skin grafting and skin flap operation, the skin grafting area survive, with satisfactory blood circulation around the skin flap area

After anaesthesia, the patient was placed supine on the fluoroscopy operating table, and a tourniquet was applied to the affected limb. The affected area was thoroughly debrided and flushed. Necrotic skin, blistered scars, and skin lacerations were removed, and the wound was thoroughly debrided. A 5 cm surgical incision was made at the front of the middle tibia, where the skin was swollen. Blood clots were removed, and the wound surface was extensively washed with weak iodophor and normal saline. An anatomical reduction was performed before the external placement of the titanium alloy distal tibia anatomical locking steel plate, which often required limited open reduction and temporary fixation with Kirschner wire. Kirschner wire fixation was also necessary for disc-shaped fractured blocks to preserve the blood supply at the fractured end and reduce soft tissue damage caused by the attachment of bone fragments. The alignment of the fracture was confirmed under C-arm fluoroscopy, and reduction was maintained. The details of patients who received the locking plate as an external fixation are in Table 1 in the supplementary file. The corresponding images for this case are shown in Fig. 1.

Appropriate locking plates (titanium alloy) were selected based on personalised principles. After confirming the correct position of the steel plate under C-arm fluoroscopy, the screws were locked one by one at the far and near ends of the fracture, with the steel plate positioned 1 cm away from the skin. To ensure the maximum "working length" of each screw, each screw was passed through both sides of the cortex as much as possible. Once the external fixation was complete, the soft tissue skin wound was repaired, and an attempt was made to close it at one stage as much as possible.

The precautions for external plate fixation are as follows:

A. Choose a relatively long plate so that there are more nail holes to choose from at both ends of the fracture; ensure that 3 to 5 locking nails are placed at each end;
B. If the fracture is segmental, two plates of single cortical screws can be placed in the middle fracture segment.
C. The plate should be as close to the skin as possible because the farther the plate is away from the bone, the lower the mechanical strength.
D. Double cortex fixation should be used as much as possible to increase stability.
E. When the stability of one plate fixation is insufficient, two plates can be used for fixation.

#### B. The combined frame external fixation group

**Fig. 2:**
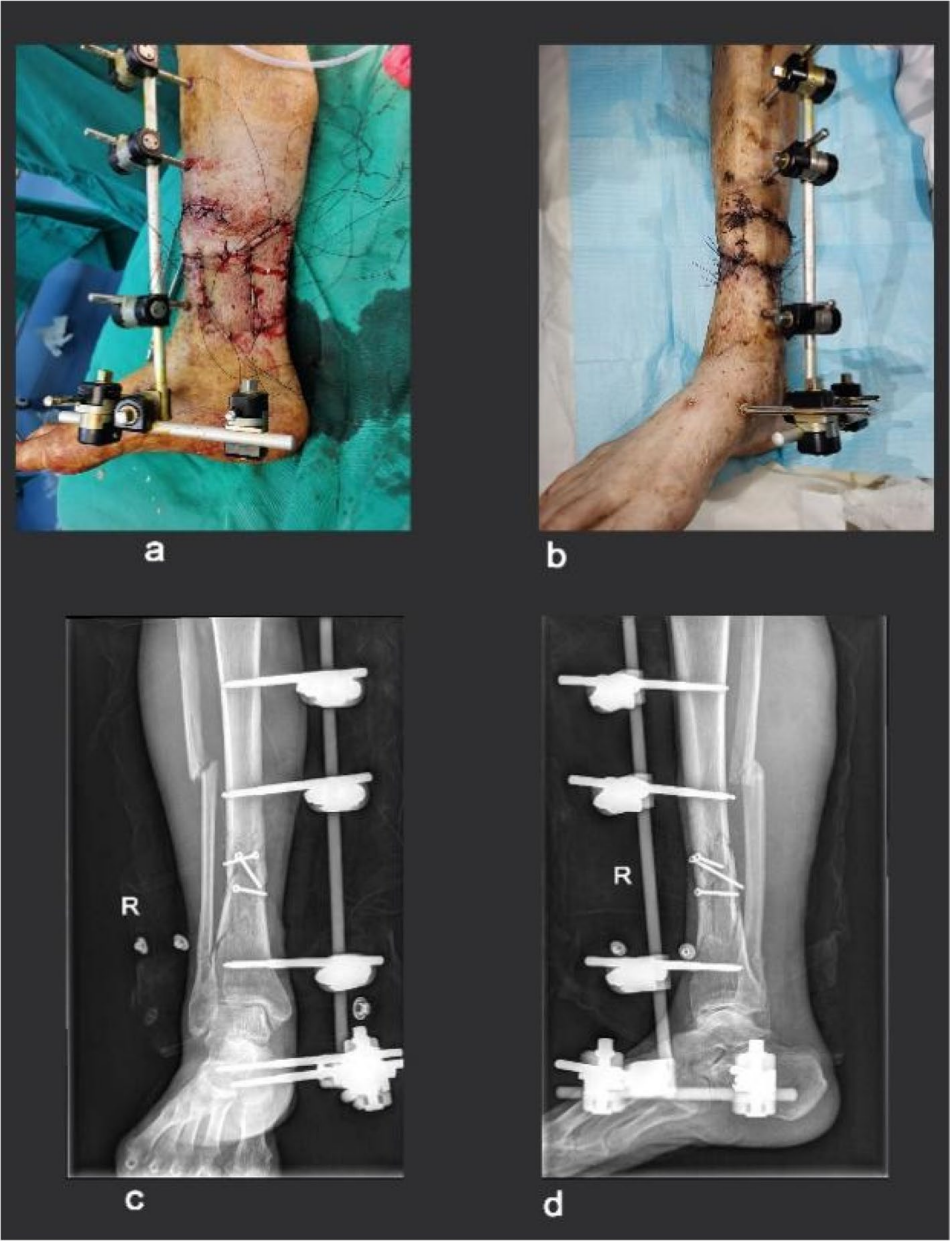
75 years old male with an open fracture of the middle and lower segment of the right tibia and fibula. **a** Day of operation, (**b**) 6.^th^-day post-operation, (**c**) and (**d**) Are Computed tomography at post-operation day 7

After anaesthesia was administered, the patient was placed in a supine position. The skin of the affected lower limb was routinely disinfected with a 1% iodine tincture. Sterile sheets were spread, the right lower limb was raised, and the airbag tourniquet on the right thigh was pressurised to 600 mmHg. Necrotic skin, blistered scars, and skin lacerations were removed, and the wound was thoroughly debrided. A 5 cm surgical incision was made at the front of the middle tibia, where the skin was swollen. Blood clots were removed, and the wound surface was extensively washed with weak iodophor and normal saline. Three external fixation pins were drilled into the proximal end of the fracture, and two external fixation pins were drilled into the distal end. The fractured end was manually reduced, and C-arm X-ray fluoroscopy confirmed the alignment of the fracture. An external fixation frame was then installed to stabilise the fractured end. A negative-pressure drainage tube was placed at the proximal end of the leg hematoma. The surgical incision through the middle bone and the skin wound of the distal tibia was sutured layer by layer with an aseptic dressing, and the tourniquet was released. Necrotic skin was covered with artificial skin and drained under negative pressure. The procedure was successful, with a good anaesthetic effect and satisfactory tourniquet performance. After the procedure, the patient was safely transferred back to the ward. The data for these patients were recorded in Table 2 in the supplementary file. The corresponding images for this case are shown in Fig. 2.

### Postoperative management

After surgery, antibiotics were intravenously injected for 48 h. We disinfected the nail with 75% alcohol in the morning and evening every day. After the wound has healed, the patient can bathe. The functional exercise was started 2–5 days after the operation. According to the fractured status of the patient, the patient is generally allowed to perform partial weight-bearing exercises 4 to 6 weeks after surgery.

### Clinical outcomes

The visual analogue scale (VAS) was used to evaluate the pain before the operation, three days later, and one-month post-operation. The fracture healing, complications, and functional recovery were recorded during the follow-up. The clinical healing time was defined as the dense callus formation at the fracture site, and the patient can walk with full weight bearing without pain. It also indicates the period during which the fixations were removed. The patient's recoveries were evaluated using the Johner-Wrush scoring system [10].

### Statistical analysis

IBM SPSS 27.0.1 software was used to analyse the data. The measurement data were compared using a t-test, while the enumeration data used the chi-square test. A *P*-value of 0.05 was considered a statistically significant difference.

### Sample characteristics

The Shapiro–Wilk test was used to evaluate the normality of the distribution of the Gustilo-Anderson (GA) classification and age between the two groups. It showed that the GA classification was approximately normally distributed between Groups A and B, with *p*-values of 0.18 and 0.14, respectively. The Shapiro–Wilk test results for the age distribution between Groups A and B show the age was normally distributed with *p*-values of 0.501 and 0.616, respectively (> 0.05), as shown in Table 1. A visual inspection of their histograms, normal Q-Q plots, and box plots showed that the age and GA classification were approximately normally distributed in both groups.

**Table 1:** Test for normality

		Fracture classification		Age	
		Group A	Group B	Group A	Group B
Shapiro–Wilk	*p*-values	0.18	0.14	0.501	0.616

An independent-sample t-test was conducted to compare the age, GA classification, Hospitalization period, and estimated cost of the Locking plate external fixation and Combined frame external fixation as shown in Table 2.

**Table 2:** Differences in age, GA classification, hospitalization period, and estimated cost between groups A and B

	Groups	Levene’s test for Equality of Variances		t-test for Equality of Means						
		F	Sig	t	df	Sig. (2 tailed)	Mean Difference	Std. Error Difference	95% Confidence Interval of the Difference	
									Lower	Upper
Age	A	7.541	0.009	0.018	26.383	0.931	0.407	4.991	-9.847	10.659
	B									
GA classification	A	0.32	0.574	-0.779	42.00	0.440	-0.259	0.332	-0.930	0.412
	B									
Hospitalization period	A	6.248	0.016	-2.309	33.112	0.027	-9.876	4.279	-18.580	-1.171
	B									
Estimated cost	A	3.399	0.072	-2.496	42	0.017	-12,476.377	4998.20	-22,563.1	-2389.59
	B									

## Results

The locking plate had 19 patients, 13 males, and six females, with a mean age of 52.6818.31 years. The combined frame external fixation had 25 patients, 22 males, and three females, with a mean age of 52.7611.67. Detailed patient characteristics for both groups can be found in Tables 1 and 2 of the supplementary files.

The research found significant variations in clinical outcomes, complications, and cost consequences between Group A and Group B. Group A had lower hospitalisation periods than Group B. Re-operation rates was also considerably lower in Group A than Group B, owing to a greater prevalence of pin-tract infections and subsequent pin loosening in the combination frame external fixator group. Complication rates were similarly lower in Group A than in Group B, with infections being the most common cause in both groups.

Furthermore, Group A showed higher functional recovery, with a high percentage of patients scoring excellent on the Johner-Wrush scale and a few scoring good. On the other hand, Group B had a comparatively smaller percentage of patients scoring excellent but a more significant proportion of good scores compared to Group A. There were not many differences in pain tolerance between the two groups. The estimated cost of both techniques was recorded and analysed with the locking averaging 26,619.69 ± 9,602.352 and the combined frame averages 39,095.64 ± 20,070.077. The patients showed good functional recovery, and none showed an unclear length of the lower limbs during the follow-up. The analysis of this result is recorded in Table 3.

**Table 3:** The outcomes of the participants

Serial No		Group A (*N* = 19)	Group B (*N* = 25)
1	Age (years)	52.68 ± 18.31	52.76 ± 11.67
2	Gender	%	
	Male	68.4	88
	Female	31.6	12
3	Hospitalization period (day)	23.68 ± 7.74	33.56 ± 19.47
4	Clinical healing period (month)	2.84 ± 0.47	3.20 ± 0.43
5	Pre-operation VAS score	7.63 ± 1.26	7.20 ± 1.12
6	Post operation day 3 VAS score	3.21 ± 0.71	3.28 ± 0.68
7	Post operation 1-month VAS score	1.16 ± 0.83	1.32 ± 1.20
8	Gustilo-Anderson classification	%	
	Type 1	15.8	12
	Type 2	47.4	40
	Type 3 A	21.1	20
	Type 3 B	10.5	24
	Type 3 C	5.3	4
9	Re-operation	%	
	Yes	26.3	48
	No	73.7	52
10	Johner-Wrush score	%	
	Excellent	84.2	56
	Good	15.5	44
11	Complication	%	
	Had complications	15.8	48
	No complication	84.2	52
12	Average estimated cost (¥)	26,619.26 ± 9,602.352	39,095.64 ± 20,070.077

## Discussion

The primary objective of this study was to assess whether locking plate external fixation could be a more effective alternative to combined frame external fixation in managing open distal tibial fractures while also considering the cost implications of both methods. To provide context for our research, we reviewed previous studies on the treatment of open distal tibial fractures [11–15]. For instance, Liang et al. conducted a retrospective examination of 34 cases using limited-reduction and bilateral-external fixators for open and comminuted mid-distal tibial fractures with damaged soft tissue. They reported osseous union in all cases after an average of 16.3 weeks. Their study included a majority of "excellent" cases (21), along with "good" (8), "fair" (4), and one "poor" case. A significant proportion (85.29%) of fractures achieved "excellent" or "good" recovery [16]. Another study by Luo et al. suggested that locked plating as an external fixator for tibial fractures could be a safe and successful procedure. However, they recommended further research to establish its superiority over standard techniques in terms of clinical and functional outcomes [17].

In a study conducted by Franz D. et al., using the gentamicin-coated nail, infection rates and total expenses for in-hospital treatment could be decreased by 75% and up to 15%, respectively. Fewer infections, fewer hospital days, and fewer re-operations may be connected with the use of antibiotic-coated IMN [18]. According to the research conducted by Bakshi et al., it was determined that the antibiotic-coated intramedullary interlocking nail (ACIIN) does not provide sufficient stability for bone defects exceeding a length of 4 cm compared to external fixation. Alternatively, the utilisation of external fixators such as Ilizarov and LRS is recommended. The study shows that the use of antibiotic-coated nails, despite their ease of use and ability to prevent pin loosening and infections, stiffened neighbouring joints more than ACIIN [3].

Our findings have significant implications for clinical practice and resource allocation in the treatment of open distal tibia fractures. Group A, which utilised the locking method, demonstrated favourable outcomes such as shorter hospitalisation periods, lower re-operation rates, and fewer complications compared to Group B, which employed the combined frame external fixator technique. These results strongly indicate the potential advantages of adopting the locking approach as the primary treatment for open distal fractures.

Shorter hospitalisation periods in Group A have important implications for reducing healthcare expenditures. They optimise the utilisation of hospital resources, minimise financial burdens on patients and healthcare systems, and improve overall efficiency. The lower re-operation rates in Group A further support the effectiveness of the locking plate technique, as it reduces the need for subsequent surgeries and related complications. On the other hand, the higher re-operation rates in Group B, which were mostly caused by pin-tract infections and loosening pins, make us worry about how safe and effective the combined frame external fixator technique is in the long run. A study by Lageju et al. reported pin-tract myiasis, an uncommon neglected wound following external fixation, emphasising the importance of wound care and risk factor avoidance in preventing this infection [19].

Even though we did everything we needed to do after surgery, we saw pin-tract infections that caused pins to come loose in patients who had the combined frame external fixation. Non-compliance with precautions in these patients may be attributed to the burden associated with the bulkiness of the fixator. Minor pin-site infections were treated with short courses of oral antibiotics without complications, while significant and deep infections necessitated pin removal and prolonged courses of antibiotics, yielding satisfactory outcomes. Mild pin-site infections were treatable with brief oral antibiotic regimens, according to a similar study by Rogers et al. involving pediatric patients with complicated distal physeal tibial shaft fractures [20]. To improve patient outcomes, further research is needed to explore infection prevention techniques associated with this procedure. Table 4 presents both groups' *P*-values and T-values of re-operation rates, clinical healing time, and complications.

**Table 4:** _Comparing the *P*-values and T-tests of the two studies_

_Serial No_		_Group A_		_Group B_	
		_*P*-values_	_t-values_	_*P*-values_	_t-values_
_1_	_Re-operation_	_0.44_	_0.79_	_0.38_	_-0.90_
_3_	_Clinical healing time_	_0.29_	_-1.11_	_0.96_	_-0.05_
_4_	_Complication_	_0.04_	_-3.39_	_0.92_	_-0.10_

The Johner-Wrush scale served as a valuable tool for assessing the impact of the locking technique on functional outcomes. The higher proportion of Group A patients with excellent scores suggests superior functional recovery. This indicates that the locking technique enhances overall recovery and quality of life compared to the combined frame and external fixator approach. Group B exhibited a lower proportion of excellent scores but a higher proportion of good scores, suggesting that while the combined frame external fixator technique may yield satisfactory functional outcomes, it falls short of the superior outcomes achieved by the locking technique. The superior functional outcomes of the locking plate can be attributed to its high flexibility and stability. This is supported by a study conducted by Blažević et al., which utilised finite element analysis to compare the stability of an external locking plate fixator with that of a conventional external fixator for extraarticular proximal tibial fractures. The results demonstrated that the external locking plate fixator exhibited greater flexibility, decreasing stiffness as the distance between the plate or rod and the bone surface increased. The study concluded that external locking plate fixation provides more flexibility than conventional external fixation and can influence secondary bone healing [21]. In the same way, Liu et al. looked at how stiff an external locking compression plate (LCP) was when it was used as an external fixator for distal tibial fractures. They got similar results, so they suggested that the distal femur locking compression plate be used to treat distal tibia fractures instead of the distal tibial locking compression plate [22].

Our cost analysis revealed that the locking plate approach (Group A) had lower estimated costs than the combined frame external fixator technique (Group B). This cost advantage, coupled with the observed clinical benefits, strengthens the case for the economic viability and cost-effectiveness of the locking plate technique in treating open distal tibial fractures. Reduced costs resulting from shorter hospitalisation periods, lower re-operation rates, and fewer complications contribute to more efficient resource allocation and potentially alleviate the financial burden on patients. Albright et al. did a study on the cost of a 30-day episode of care for high-energy tibial plateau fractures. They found that the most important cost factor was the choice of external fixation components [23]. Our study's findings in Tables 3 and 4 in the supplementary file corroborate this, showing that each component of the combined frame external fixation is nearly equivalent to the cost of the entire locking plate. Subsidies on the locking plate may account for this discrepancy. The high rate of reoperation in patients treated with the combined frame external fixation resulted in a longer hospitalisation period and thus a higher cost.

## Strengths and limitations

One of the strengths of this study is that all surgeries were performed in a single centre by the same surgical team, ensuring consistency in the surgical approach. However, this also introduces a limitation, as it prevents us from determining whether the clinical outcomes could be associated with the skills of the operating team. Further studies comparing different surgical teams could help elucidate this aspect. The occurrence of pin-tract infections and subsequent pin loosening in patients from Group B raises concerns about the safety and long-term efficacy of the combined frame external fixator technique. Further research on infection prevention techniques associated with this procedure is warranted to enhance patient outcomes.

Although this study effectively compared and analysed the two groups, its small sample size is a drawback. Future studies with larger sample sizes and extended follow-up periods will be necessary to confirm these findings. Additionally, the estimated costs of both groups are preliminary, and additional studies are needed to determine the actual costs of both techniques.

## Conclusion

In conclusion, the results of this study suggest that the locking plate technique (Group A) is the optimal treatment strategy for open distal tibia fractures. The results indicate potential benefits such as shorter hospitalisation periods, lower re-operation rates, and fewer complications for patients, healthcare systems, and resource allocation. The excellent functional recovery observed in Group A further supports the usefulness of the locking plate technique in improving overall recovery and patient quality of life. Furthermore, the cost analysis emphasises the economic feasibility and cost-effectiveness of the locking plate technique, highlighting its potential to optimise healthcare resources for open distal tibia fractures. Practical implementation of these findings, along with further research, has the potential to enhance patient outcomes and guide evidence-based orthopaedic surgical procedures.

### Supplementary Information

**Additional file 1: Table 1.** Group A (Team Locking Plate) patients’ characteristics. **Table 2.** Group B (The patients that received the usual combined external fixation system-external fixation brackets) patients’ characteristics. **Table 3.** Group A (Team Locking plate) estimated expenditure. **Table 4.** Group B (Team Combined frame external fixator) estimated expenditure.

## Data Availability

All data generated or analyzed during this study are attached as a supplementary file.

## Abbreviations

LCP: Locking compression plate
RTA: Road traffic accident
PTI: Pint-tract infection
