# Supplementary material for: Comparative analysis of external locking plate and combined frame external fixator for open distal tibial fractures: a comprehensive assessment of clinical outcomes and financial implications
Source: BMC Musculoskelet Disord. 2023 Dec 11;24:962. doi: 10.1186/s12891-023-07097-z (PMC10712054; doi:10.1186/s12891-023-07097-z)
Supplement: Supplementary file 1 — Additional file 1: Table 1. Group A (Team Locking Plate) patients’ characteristics. Table 2. Group B (The patients that received the usual combined external fixation system-external fixation brackets) patients’ characteristics. Table 3. Group A (Team Locking plate) estimated expenditure. Table 4. Group B (Team Combined frame external fixator) estimated expenditure. [file 12891_2023_7097_MOESM1_ESM.docx]

| ***Table 1*: Group A (Team Locking Plate) patients’ characteristics** | | | | | | | | | | | | | |
| --- | --- | --- | --- | --- | --- | --- | --- | --- | --- | --- | --- | --- | --- |
| Case | Age (years) | Gender | Fracture side | Cause of injury | Hospitalization period (days) | Re-operation | Clinical Healing time (months) | Complication | Johner-Wrush score | Gustilo-Anderson classification | VAS Score | | |
|  |  |  |  |  |  |  |  |  |  |  | Pre-operation | Post operation three days | Post operation one month |
| 1 | 44 | Male | Left | RTA | 30 | Yes | 3 | PTI | Excellent | 3C | 10 | 2 | 2 |
| 2 | 68 | Male | Right | RTA | 28 | No | 2.5 | No | Excellent | 3A | 7 | 3 | 1 |
| 3 | 55 | Female | Left | Fall from height | 26 | No | 3 | No | Excellent | 3A | 8 | 4 | 1 |
| 4 | 31 | Male | Left | Sport injury | 18 | No | 2 | No | Excellent | 2 | 7 | 3 | 2 |
| 5 | 28 | Female | Right | Crush injury | 23 | Yes | 2.5 | No | Excellent | 2 | 8 | 2 | 1 |
| 6 | 43 | Male | Left | RTA | 45 | Yes | 3 | No | Excellent | 3A | 9 | 4 | 0 |
| 7 | 74 | Male | Right | Fall from height | 25 | No | 3 | No | Excellent | 2 | 8 | 3 | 1 |
| 8 | 32 | Female | Left | RTA | 24 | No | 2.5 | No | Excellent | 2 | 9 | 4 | 0 |
| 9 | 20 | Male | Left | Sport injury | 30 | No | 2 | No | Excellent | 3A | 8 | 4 | 2 |
| 10 | 70 | Male | Left | RTA | 29 | Yes | 3 | Skin necrosis  PTI | Good | 3B | 10 | 4 | 3 |
| 11 | 48 | Male | Right | Other | 22 | No | 3 | No | Excellent | 2 | 7 | 4 | 1 |
| 12 | 52 | Male | Left | RTA | 13 | No | 3 | No | Excellent | 2 | 6 | 3 | 0 |
| 13 | 34 | Female | Right | Crush injury | 17 | No | 2.5 | No | Excellent | 1 | 6 | 3 | 1 |
| 14 | 70 | Female | Left | Fall from height | 14 | No | 3 | No | Excellent | 1 | 6 | 4 | 2 |
| 15 | 75 | Male | Left | Fall from height | 25 | Yes | 3 | No | Excellent | 2 | 7 | 3 | 1 |
| 16 | 48 | Male | Left | RTA | 30 | No | 2.5 | No | Excellent | 3A | 8 | 5 | 1 |
| 17 | 61 | Female | Left | RTA | 13 | No | 3 | No | Excellent | 2 | 8 | 3 | 0 |
| 18 | 82 | Male | Left | Other | 18 | No | 4 | PTI | Good | 2 | 7 | 2 | 1 |
| 19 | 66 | Male | Right | Fall from height | 30 | No | 3.5 | No | Excellent | 2 | 2 | 3 | 2 |
| Note: RTA=Road traffic accident, PTI=Pin-tract infection | | | | | | | | | | | | | |

| ***Table 2*: Group B (The patients that received the usual combined external fixation system-external fixation brackets) patients’ characteristics** | | | | | | | | | | | | | |
| --- | --- | --- | --- | --- | --- | --- | --- | --- | --- | --- | --- | --- | --- |
| Case | Age  (years) | Gender | Fracture  Side | Cause of injury | Hospitalization  Period  (days) | Re-operation | Clinical healing  Time  (month) | Complication | Johner-Wrush  score | Gustilo-Anderson  Classification | VAS Score | | |
|  |  |  |  |  |  |  |  |  |  |  | Pre-operation | Post operation three days | Post operation 1month |
| 1 | 75 | Male | Right | RTA | 25 | No | 3 | No | Excellent | 2 | 7 | 3 | 0 |
| 2 | 72 | Male | Left | RTA | 28 | Yes | 3 | No | Good | 3A | 8 | 3 | 3 |
| 3 | 68 | Male | Left | Other | 36 | Yes | 3 | No | Excellent | 3B | 9 | 4 | 2 |
| 4 | 57 | Male | Right | Other | 14 | No | 3 | No | Excellent | 2 | 7 | 2 | 0 |
| 5 | 54 | Male | Left | RTA | 60 | No | 3 | Mal-union | Good | 3C | 9 | 3 | 2 |
| 6 | 57 | Male | Left | RTA | 40 | Yes | 3 | PTI | Good | 3A | 7 | 4 | 3 |
| 7 | 50 | Male | Right | Other | 11 | No | 3 | No | Excellent | 1 | 6 | 4 | 0 |
| 8 | 33 | Male | Left | RTA | 68 | No | 4 | Skin necrosis, PTI | Good | 3B | 8 | 4 | 2 |
| 9 | 47 | Male | Both | RTA | 55 | Yes | 4 | PTI | Good | 3B | 10 | 3 | 2 |
| 10 | 60 | Male | Right | RTA | 38 | No | 3 | No | Excellent | 2 | 6 | 2 | 0 |
| 11 | 54 | Male | Right | RTA | 91 | Yes | 4 | Mal-union | Good | 3B | 8 | 3 | 3 |
| 12 | 53 | Female | Left | RTA | 29 | No | 3 | No | Excellent | 2 | 6 | 3 | 0 |
| 13 | 47 | Female | Right | RTA | 31 | Yes | 3 | Mal-union | Good | 2 | 7 | 4 | 2 |
| 14 | 62 | Male | Right | Other | 15 | No | 3 | No | Excellent | 2 | 6 | 3 | 1 |
| 15 | 67 | Male | Right | RTA | 30 | Yes | 3.5 | PTI | Good | 2 | 6 | 3 | 1 |
| 16 | 51 | Female | Left | RTA | 30 | No | 3 | No | Excellent | 3A | 8 | 4 | 1 |
| 17 | 47 | Male | Right | Other | 25 | Yes | 3 | PTI | Good | 3A | 8 | 3 | 1 |
| 18 | 45 | Male | Left | Other | 30 | Yes | 3 | Mal-union | Excellent | 2 | 6 | 4 | 2 |
| 19 | 58 | Male | Left | RTA | 35 | Yes | 4 | Skin necrosis, PTI | Good | 3B | 7 | 4 | 3 |
| 20 | 29 | Male | Both | RTA | 51 | Yes | 4 | Skin necrosis, PTI, Mal-union | Good | 3B | 7 | 4 | 2 |
| 21 | 56 | Male | Right | RTA | 35 | Yes | 3 | PTI | Excellent | 3A | 7 | 3 | 1 |
| 22 | 57 | Male | Right | RTA | 30 | No | 3 | No | Excellent | 2 | 6 | 4 | 1 |
| 23 | 44 | Male | Right | RTA | 12 | NO | 3 | No | Excellent | 1 | 6 | 3 | 0 |
| 24 | 45 | Male | Right | RTA | 8 | NO | 3 | No | Excellent | 1 | 7 | 3 | 1 |
| 25 | 31 | Male | Left | Other | 12 | No | 2.5 | No | Excellent | 2 | 8 | 2 | 0 |

Note: RTA=Road traffic accident, PTI=Pint-tract infection

| ***Table 3*: Group A (Team Locking plate) estimated expenditure** | | | | | | | | | | | | | | |
| --- | --- | --- | --- | --- | --- | --- | --- | --- | --- | --- | --- | --- | --- | --- |
| Case | Material cost (¥) | Surgical and Anesthesia fees (¥) | Consultation fee (¥) | Bed fee/day (¥) | Hospitalization  period(days) | Total bed fee (¥) | Nursing fee/day (¥) | Total nursing fee (¥) | Medication fee (¥) | CT scan fee (¥) | Medical examination fees (¥) | | | Total Cost (¥) |
|  |  |  |  |  |  |  |  |  |  |  | Laboratory fee (¥) | ECG fee (¥) | Other radiology fees (¥) |  |
| 1 | 450 | 22,848 | 296 | 42 | 30 | 1,260 | 24 | 720 | 12,425 | 2,156 | 8,988 | 37 | 814 | 49,994 |
| 2 | 450 | 20,727 | 407 | 42 | 28 | 1,176 | 24 | 672 | 6,010 | 1,298 | 4,741 | 74 | 546 | 36,101 |
| 3 | 450 | 4,700 | 330 | 42 | 26 | 1,092 | 24 | 624 | 5,500 | 1,800 | 4,600 | 37 | 420 | 19,553 |
| 4 | 450 | 16,000 | 296 | 42 | 18 | 756 | 24 | 432 | 4,400 | 2,200 | 5,200 | 37 | 720 | 30,491 |
| 5 | 450 | 14,848 | 330 | 42 | 23 | 966 | 24 | 552 | 8,000 | 2,156 | 7,300 | 74 | 530 | 35,206 |
| 6 | 450 | 12,848 | 296 | 42 | 45 | 1,890 | 24 | 1,080 | 11,200 | 1,298 | 3,600 | 37 | 620 | 33,319 |
| 7 | 450 | 8,300 | 407 | 42 | 25 | 1,050 | 24 | 600 | 6,000 | 1,800 | 5,200 | 74 | 380 | 24,261 |
| 8 | 450 | 9,400 | 290 | 42 | 24 | 1,008 | 24 | 576 | 7,200 | 2,156 | 4,741 | 37 | 546 | 26,404 |
| 9 | 450 | 8,951 | 407 | 42 | 30 | 1,260 | 24 | 720 | 8000 | 1,800 | 5,600 | 74 | 546 | 27,808 |
| 10 | 450 | 23,000 | 390 | 42 | 29 | 1,218 | 24 | 696 | 4,900 | 2,156 | 3,000 | 37 | 730 | 36,577 |
| 11 | 450 | 8,890 | 330 | 42 | 22 | 924 | 24 | 528 | 12,620 | 2,200 | 7,700 | 74 | 800 | 34,516 |
| 12 | 450 | 4,620 | 290 | 42 | 13 | 546 | 24 | 312 | 9,647 | 981 | 8,500 | 74 | 600 | 26,000 |
| 13 | 450 | 6,400 | 395 | 42 | 17 | 714 | 24 | 408 | 8,577 | 790 | 6,675 | 37 | 740 | 25,186 |
| 14 | 450 | 9,600 | 296 | 42 | 14 | 588 | 24 | 336 | 2,800 | 689 | 4,356 | 37 | 340 | 19,492 |
| 15 | 450 | 5,000 | 340 | 42 | 25 | 1,050 | 24 | 600 | 2,240 | 698 | 583 | 37 | 933 | 11,931 |
| 16 | 450 | 6,600 | 407 | 42 | 30 | 1,260 | 24 | 720 | 6,900 | 796 | 3,189 | 74 | 980 | 21,376 |
| 17 | 450 | 5,043 | 360 | 42 | 13 | 546 | 24 | 312 | 2,302 | 878 | 1,668 | 37 | 412 | 12,008 |
| 18 | 450 | 10,000 | 400 | 42 | 18 | 756 | 24 | 432 | 2,600 | 789 | 2,600 | 74 | 470 | 18,571 |
| 19 | 450 | 8,800 | 395 | 42 | 30 | 1,260 | 24 | 720 | 1,790 | 980 | 1,980 | 37 | 560 | 16,972 |
| Note: CT=Computerized Tomography, ECG=Electrocardiogram. Material cost refers to the cost of the implants and their components. | | | | | | | | | | | | | | |

| ***Table 4*: Group B (Team Combined frame external fixator) estimated expenditure** | | | | | | | | | | | | | | |
| --- | --- | --- | --- | --- | --- | --- | --- | --- | --- | --- | --- | --- | --- | --- |
| Case | Material cost (¥) | Surgical and Anesthesia fees (¥) | Consultation fee (¥) | Admission fee/day (¥) | Hospitalization  period(days) | Total admission fee (¥) | Nursing fee/day (¥) | Total nursing fee (¥) | Medication fee (¥) | CT scan fee (¥) | Medical examination fees (¥) | | | Total Cost (¥) |
|  |  |  |  |  |  |  |  |  |  |  | Laboratory fee (¥) | ECG fee (¥) | Other radiology fees (¥) |  |
| 1 | 9,000 | 4,800 | 300 | 42 | 25 | 1,050 | 24 | 600 | 4,300 | 2,120 | 3,500 | 37 | 540 | 26,247 |
| 2 | 12,000 | 20,000 | 290 | 42 | 28 | 1,175 | 24 | 672 | 5,780 | 970 | 3,250 | 37 | 890 | 45,064 |
| 3 | 15,750 | 20,500 | 296 | 42 | 36 | 1,512 | 24 | 1,224 | 6,540 | 886 | 4,000 | 37 | 670 | 51,415 |
| 4 | 9,000 | 5,600 | 420 | 42 | 14 | 588 | 24 | 336 | 2,500 | 3,200 | 3,700 | 37 | 489 | 25,870 |
| 5 | 40,827 | 42,848 | 296 | 42 | 60 | 2,520 | 24 | 1,440 | 12,426 | 2,156 | 8,780 | 74 | 820 | 112,187 |
| 6 | 11,250 | 18,900 | 407 | 42 | 40 | 1,680 | 24 | 960 | 4,150 | 1,050 | 9,700 | 74 | 980 | 49,151 |
| 7 | 9,000 | 4,625 | 79 | 42 | 11 | 462 | 24 | 264 | 780 | 2,700 | 6,900 | 37 | 490 | 25,337 |
| 8 | 15,000 | 4,400 | 350 | 42 | 68 | 2,856 | 24 | 1,44632 | 3,700 | 2,400 | 12,400 | 74 | 1,200 | 44,012 |
| 9 | 15,000 | 20,000 | 400 | 42 | 55 | 2,310 | 24 | 1,320 | 2,400 | 2,780 | 10,350 | 74 | 998 | 55,632 |
| 10 | 9,000 | 5,250 | 260 | 42 | 38 | 1,596 | 24 | 912 | 1,900 | 540 | 4,900 | 37 | 575 | 24,970 |
| 11 | 15,000 | 18,750 | 198 | 42 | 91 | 3,822 | 24 | 2,184 | 5,800 | 680 | 14,500 | 74 | 2,700 | 63,708 |
| 12 | 9,000 | 4,600 | 305 | 42 | 29 | 1,218 | 24 | 696 | 1,350 | 615 | 2,700 | 37 | 690 | 21,211 |
| 13 | 9,000 | 20,000 | 200 | 42 | 31 | 1,302 | 24 | 744 | 1,540 | 981 | 2,980 | 37 | 550 | 37,334 |
| 14 | 9,000 | 6,250 | 340 | 42 | 15 | 630 | 24 | 360 | 890 | 876 | 980 | 37 | 355 | 19,718 |
| 15 | 9,000 | 17,560 | 120 | 42 | 30 | 1,260 | 24 | 720 | 1,895 | 1,300 | 3,400 | 37 | 470 | 35,720 |
| 16 | 11,250 | 15,400 | 209 | 42 | 30 | 1,260 | 24 | 720 | 1,970 | 1,120 | 4,200 | 37 | 530 | 36,696 |
| 17 | 11,250 | 18,300 | 296 | 42 | 25 | 1,050 | 24 | 600 | 1,090 | 1,230 | 3,700 | 37 | 589 | 38,142 |
| 18 | 9,750 | 12,790 | 296 | 42 | 30 | 1,260 | 24 | 720 | 1,600 | 600 | 1,780 | 37 | 430 | 29,263 |
| 19 | 15,000 | 21,000 | 210 | 42 | 35 | 1,470 | 24 | 840 | 2,450 | 1,275 | 3,975 | 74 | 566 | 46,860 |
| 20 | 15,750 | 20,000 | 390 | 42 | 51 | 2,142 | 24 | 1,224 | 4,800 | 1,750 | 8,895 | 74 | 890 | 55,915 |
| 21 | 11,250 | 16,860 | 365 | 42 | 35 | 1,470 | 24 | 840 | 3,000 | 2,300 | 3,670 | 74 | 675 | 40,504 |
| 22 | 9,000 | 17,700 | 245 | 42 | 30 | 1,260 | 24 | 720 | 2,600 | 675 | 2,880 | 37 | 780 | 35,897 |
| 23 | 9,000 | 8,000 | 220 | 42 | 12 | 504 | 24 | 288 | 790 | 850 | 720 | 37 | 240 | 20,649 |
| 4 | 9,000 | 5,500 | 245 | 42 | 8 | 336 | 24 | 192 | 600 | 670 | 530 | 37 | 240 | 17,350 |
| 25 | 9,000 | 6,000 | 298 | 42 | 12 | 504 | 24 | 288 | 712 | 730 | 660 | 37 | 310 | 18,539 |
| Note: CT=Computerized Tomography, ECG=Electrocardiogram. Material cost refers to the cost of the implants and their components. | | | | | | | | | | | | | | |
